# Transactions between self-esteem and perceived conflict in romantic relationships: A 5-year longitudinal study

**DOI:** 10.1371/journal.pone.0248620

**Published:** 2021-04-12

**Authors:** Julia Richter, Christine Finn

**Affiliations:** 1 Department of Psychology, Bielefeld University, Bielefeld, Germany; 2 Department of Psychology, Friedrich-Schiller University, Jena, Germany; Universitat zu Lubeck Institut fur Psychologie I, GERMANY

## Abstract

Self-esteem has been shown to be both predictive of and predicted by characteristics of romantic relationships. While there is an increasing number of studies yielding support for reciprocal influences between self-esteem and perceived conflict in romantic relationships, longitudinal transactions between these constructs from both partners’ perspectives have not been studied systematically to date. Our aim was to close this gap. To that end, we examined the transactional and longitudinal interplay between self-esteem and perceived relationship conflict in continuing romantic couples from a dyadic perspective. Our sample consisted of *N* = 1,093 young adult female–male relationships from the German Family Panel. Individuals’ self-esteem, perceived conflict frequency, and their perceptions of their partners’ dysfunctional conflict styles (i.e., unconstructive behavior, withdrawal) were examined annually throughout a time span of five years. Based on dyadic bivariate latent change models, we tested our assumption that self-esteem and aspects of perceived relationship conflict are negatively interrelated within individuals and between partners both within and across time. We found one actor effect of self-esteem on changes in unconstructive behavior above and beyond initial unconstructive behavior levels, supporting self-broadcasting perspectives. Moreover, we found strong support for sociometer perspectives. Actor effects highlighted the importance of perceived conflict frequency for subsequent self-esteem changes. In addition, perceived conflict styles affected both partners’ self-esteem. The results imply that perceiving conflict is a between-person process, and might be more important for the development of self-esteem than vice versa.

## Introduction

Global self-esteem refers to an individual’s overall self-evaluation of being a person of worth [1]. It is moderately stable across time [2, 3]. From the perspective of dynamic transactionism [4] and according to the interdependence theory [5], self-esteem is not independent of the individual’s social environment, but directly interwoven with social relationships and experiences [6, 7]. Since romantic relationships might be the closest form of social relationships that adults in Western societies can have [8], continuing romantic relationships seem to be a perfect context for studying personality–relationship transactions [6]. Focusing much of its attention on the forming and succeeding of partnerships, previous research has shown that self-esteem is a strong predictor of relationship characteristics [6, 9, 10]. Moreover, having or establishing a partnership can have positive effects on self-esteem changes [6, 11, 12]. These studies showed that self-esteem and positive relationship characteristics foster a mutual development. However, romantic relationships comprise multiple aspects [11] of both positive and negative evaluative character. To understand partner dynamics more extensively, it is necessary to also investigate negative self-esteem–relationship dynamics.

### Conflict in romantic relationships

One rather negative relationship characteristic is relationship conflict [12]. Conflict is defined as “the perception of contention or disaccord” [13, p. 13] between at least two persons. As per definition, conflict occurs in relationships (e.g., romantic partnerships, parent–child relationships). While relationship conflict might not generally be detrimental, high *conflict frequency* may indicate substantive imbalances in a relationship. In fact, conflict and satisfaction in romantic relationships seem to counteract each other [14–16]. Negative behavior during disagreements, i.e., *conflict styles*, can in part explain a detrimental effect of conflict on relationship satisfaction [17]. In a study by Bertoni and Bodenmann [18], the authors compared satisfied with dissatisfied couples regarding their conflict styles. Respondents reported their tendency for offence, compromise (i.e., constructive behavior), avoidance (i.e., withdrawal), and violence during conflicts. Satisfied couples showed more functional conflict styles (i.e., less dysfunctional conflict styles) in comparison to dissatisfied couples. Stanley, Markman, and Whitton [16] reported similar results. In their study, negative conflict styles (e.g., withdrawal) were associated with lower relationship satisfaction and with more ruminations about divorce. These studies underscore the relevance of conflict frequency and conflict styles (e.g., constructive behavior and withdrawal) for partner dynamics in romantic relationships.

### Sociometer and self-broadcasting perspectives on self-esteem dynamics in romantic relationships

As described below, both self-broadcasting and sociometer perspectives can explain why regularly experiencing conflict as well as perceiving uncompromising or rejecting partner behavior during a fight might be negatively associated to self-esteem [7, 19, 20]. Surprisingly, however, only few studies to date have explicitly investigated longitudinal associations between self-esteem and perceived conflict in dating couples from both partners’ perspectives [21], and none of these studies have examined actor–partner (i.e., dyadic) transactions over the course of several years. With our study, we aim to fill this gap by examining dyadic transactions between self-esteem and multiple indicators of perceived relationship conflict in continuing romantic relationships throughout a time span of five years. In the following paragraphs, we will outline how sociometer and self-broadcasting perspectives predict associations between self-esteem and relationship conflict.

#### Sociometer perspectives

From the sociometer perspective, self-esteem is dependent on social embeddedness. More specifically, self-esteem is assumed to monitor an individual’s social inclusion as a prerequisite for survival and reproduction [22]. Low self-esteem is regarded as an indicator of rejection, whereas high self-esteem is regarded as the result of social success in terms of approving social reactions [21, 23]. Romantic relationships in Western cultures are one of the rare relationships that cannot only increase survival by (possibly) being of supportive nature, but also especially serve the goal to establish a family. Given that one basic assumption of the sociometer perspective is that self-esteem serves the ultimate goal of reproduction, romantic relationships should be one of the best contexts to study this perspective. In line with this, Knee and colleagues [19] argued that successfully navigating romantic relationships plays a leading role for an individual’s self-esteem. In one of their studies, they examined self-esteem fluctuations on days after perceived positive and negative relationship events. Across all individuals, self-esteem was lower after perceived rejection situations than after positive situations [19]. Similarly, Murray, Griffin, Rose, and Bellavia [20] reported that perceiving or fearing disapproval and rejection in a relationship predicted decreases in self-esteem. This effect especially manifested in low self-esteem individuals, who were indeed chronically less positively regarded by their partners than high self-esteem individuals [20]. These studies support the assumption that changes in self-esteem may be triggered by social experiences of disapproval and rejection within romantic relationships.

In the following, we will introduce the fictitious couple Jenny and James to illustrate the complex interplay between self-esteem and conflict in a dyadic context. The sociometer perspective can help explain several scenarios regarding their interactions. First, if Jenny and James perceive multiple conflict situations with each other, this could lead both of them to feel rejected, leading to decreases in both their self-esteem. Second, perceiving his partner Jenny being withdrawing or unwilling to compromise during a fight might lead James to think that she rejects him, which decreases his self-esteem. And third, if James constantly perceives and reports that Jenny shows dysfunctional conflict behavior, this might tell something about James’ devaluation of Jenny. Put differently, James might not appreciate Jenny’s behavior, which in turn leads her to feel disapproved, and eventually decreases her self-esteem.

#### Self-broadcasting perspectives

The self-broadcasting perspective posits that individuals behave in accordance with their self-evaluations, thus evoking congruent reactions in their social encounters [24]. Low self-esteem individuals are assumed to chronically strive for approval [20]. At the same time, they seem to underestimate their partner’s regard as a consequence of a heightened attention towards hostile partner signals in the sense of chronically high rejection sensitivity [25]. Chronically high rejection sensitivity is defined as “the disposition to anxiously expect, readily perceive, and overreact to rejection” [26, p. 545]. In line with this, individuals with low self-esteem and high rejection sensitivity have been found to underestimate their partner’s satisfaction [20, 27], and to be more sensitive to actual rejection behavior by the partner [20, 28]. Additionally, low self-esteem individuals seem to distance themselves from their partner (i.e., avoidance) to minimize the risk of rejection, whereas high self-esteem individuals seem to seek closeness, even after conflict situations [14]. Another consequence from the dilemma in which low self-esteem individuals find themselves is that their increased fear of rejection might cause them to engage in dysfunctional behavior during conflicts [15, 20]. Possibly as a consequence, partners of women afraid of being rejected are indeed more rejecting [26].

In accordance with self-broadcasting perspectives, self-esteem has been found to affect self-perceived popularity among peers [29, 30]. In romantic relationships, small positive effects of self-esteem on relationship satisfaction changes have been reported [25, 31]. In line with this, Murray and colleagues [20] found that one individual’s sensitivity to rejection spilled over to their partner, leading their partner’s relationship satisfaction to decrease over the year. As fear of rejection is associated to self-esteem [27], this finding might indicate a spill-over effect of self-esteem on the partner’s satisfaction.

From self-broadcasting perspectives, several scenarios are conceivable regarding the interplay between self-esteem and conflict in our fictitious couple. First, James having low self-esteem might perceive more negative conflict behavior in his partner Jenny than a high self-esteem individual would, independently of Jenny’s actual behavior. Second, James might distance himself from Jenny. This could eventually lead Jenny (the partner of a low self-esteem individual) to perceive rejection behavior in James, mirroring his attempt for self-protection. Third, James’ fear of rejection, triggered by his low self-esteem, might lead him to act less constructively during conflicts, evoking rejection behavior in Jenny. That is, Jenny would eventually really come to be disapproving of James, possibly as a cost of his unconstructive conflict behavior.

## The present study

While some of the aforementioned studies provide valuable information on the association of self-esteem and experiences of disapproval or rejection in relationships, none of these studies have examined long-term transactions between self-esteem and perceived conflict across multiple occasions from a dyadic perspective. With our study, we aim to broaden the knowledge on self-esteem changes in accordance with perceptions of uncompromising and rejecting partner behavior. To that end, we examined self-esteem–conflict transactions in continuing romantic female–male couples across a five-year time span using a dyadic approach. A dyadic longitudinal study with continuing partnerships is appropriate because research has shown that relationship-specific characteristics and interactions are best portrayed by the perspectives of both partners in a dyadic interplay [6, 32].

Using dyadic bivariate latent-change models (following the procedure by [6]), we examined long-term associations between both partners’ self-esteem and perceived conflict frequency as well as perceived dysfunctional conflict styles in terms of the partner’s unconstructive behavior (i.e., the absence of constructive behavior) and withdrawal. More specifically, including both partners’ self-esteem and perceived conflict annually over a time span of five years in dyadic bivariate latent-change models enabled us to examine *level-change effects* to test both sociometer and self-broadcasting perspectives. Level-change effects of the level of conflict on subsequent change in self-esteem correspond to the sociometer perspective, while level-change effects of the self-esteem level on subsequent change in conflict correspond to self-broadcasting perspectives. *Change-change effects* indicate an effect of previous changes in one construct on subsequent changes in another construct. While the effect of previous changes in conflict on subsequent self-esteem changes corresponds to sociometer effects, the opposite is true for self-broadcasting effects. Moreover, change-change effects make it possible to examine bidirectional dynamics over longer time periods. Our approach to capture self-esteem–relationship transactions in a stable social context from both partners’ perspectives made it possible to examine these effects within individuals (*actor effects*) and between partners (*partner effects*).

### Hypotheses

We examined the assumption that self-esteem and perceived relationship conflict are negatively intertwined within individuals (*actor effects*) and between partners (*partner effects*) both within and across time. In line with the sociometer and self-broadcasting perspectives, we had specific hypotheses, which we will first explain in general, before associating them with the specific paths investigated via the dyadic longitudinal model approach.

#### Predictions on sociometer perspectives

Based on the sociometer perspective, we argue that perceiving withdrawal or unwillingness to compromise in a partnership should predict subsequent decreases in individuals’ self-esteem. Perceiving the partner lacking constructive conflict behavior (here termed as unconstructive behavior) and withdrawal during a fight seem to be obvious candidates to evoke perceived disapproval and rejection in individuals. We assumed that higher perceived conflict frequency in general might have the same effect. Thus, individuals who perceive more overall conflict and who perceive their partner showing more dysfunctional behavior during a fight should show stronger decreases in self-esteem (actor effect) than individuals who perceive less (dysfunctional) conflict. Moreover, we expected this effect to spill over to the partner, also leading to a subsequent self-esteem decrease in those individuals showing uncompromising or withdrawing conflict behavior (partner effect).

*Level-change effects supporting sociometer hypotheses*. A major strength of the current study is that level-change effects are not mere reflections of pre-post associations, but are specifically able to indicate whether the level of one construct has an influence on the change of another construct after controlling for the initial level of the second construct. If Jenny constantly perceives conflict in her relationship or James is little assuring during a fight (unconstructive behavior, withdrawal), this might increase feelings of disapproval and insecurity in Jenny in the long run. While we acknowledge that low self-esteem individuals might be particularly sensitive to such processes, we assume that constantly high levels of conflict may have the potential to erode self-esteem in most couples (*actor level-change effect*) [19].

Low self-esteem individuals have been shown to be less positively regarded by their partners [20]. Consequently, we expect that low partner regard would lead to subsequent decreases in self-esteem (*partner level-change effect*). That is, if James constantly perceives and reports that Jenny shows dysfunctional conflict behavior, this might be a hint on James’ potential devaluation of her, and Jenny’s self-esteem might decrease in reaction to this, too–opposite to the processes that have been found for high regard [21].

#### Predictions on self-broadcasting perspectives

In line with the self-broadcasting perspective, we argue that low self-esteem predicts a stronger perception of uncompromising or rejecting partner behaviors (actor effect). Moreover, we expected low self-esteem individuals to report more conflict (actor effect), to behave less constructively during a fight, and to show more withdrawal. From a dyadic perspective, we expected partners of low self-esteem individuals to report more conflict in general, and to report more unconstructive and withdrawal tendencies in their partner (partner effect).

*Level-change effects supporting self-broadcasting hypotheses*. In accordance with self-broadcasting perspectives, we expected self-esteem to be negatively associated with subsequent changes in perceived conflict frequency and dysfunctional conflict behavior. Put differently, low self-esteem individuals should report higher increases in their perception of arguing frequently (high conflict frequency) and/or in their perception of little assurance by their partner during a fight (unconstructive behavior, withdrawal). For example, James’ low self-esteem might lead him to increasingly perceive conflict with Jenny over time, and to increasingly perceive her behavior during a fight as dysfunctional (actor level-change effect) [20].

Previous work has shown that dysfunctional behavior arises situationally due to feelings of rejection, with low self-esteem individuals being especially prone to experience these feelings [33]. We assumed that this process might lead the partner of a low self-esteem individual to report more negative conflict behavior in their partner in the long run (partner level-change effect). Put differently, James’ low self-esteem might subsequently increase Jenny’s perception of their conflict frequency or her perception of his dysfunctional conflict behavior (i.e., unconstructive behavior, withdrawal) during a fight.

#### Predictions on change-change effects

Change-change effects denote longitudinal transactions between two constructs after controlling for their initial levels [34]. For example, change in perceived conflict frequency might predict subsequent change in self-esteem. While each change-change effect by itself might be subsumed under one certain perspective (e.g., the change-change effect of conflict frequency on self-esteem would belong to the sociometer perspective), we argue that it is most appropriate to interpret change-change effects from both directions at the same time. This way, they can inform us about bidirectional influences between multiple measurement occasions, making the interpretation of longitudinal dynamic transactions between two constructs possible. Support for the expectation of longitudinal transactions comes from accumulating negative circles of disapproving self-evaluations and relationship experiences [20].

With regard to change-change effects, we expected that an increase (decrease) in perceived conflict behavior might lead to a later decrease (increase) in self-esteem and vice versa (change-change effects) [20, 35]. That is, increases in James’ perceived conflict frequency and/or perceived partner dysfunctional conflict behavior across one year might lead to decreases in his self-esteem during the subsequent year, while decreases in James’ self-esteem between T1–T2 should predict increases in his perceived conflict frequency and/or perceived partner dysfunctional conflict behavior between T2–T3 (compared to an average person). Based on assumptions of mutual dynamics between partners [4, 5], we predicted these effects both within individuals (*actor change-change effects*) and between partners (*partner change-change effects*). Please note, however, that floor and ceiling effects are able to distort change-change effects.

## Materials and methods

For the present study, we used data from the German Family Panel (pairfam) [36]. Pairfam is a representative longitudinal study, which incorporates a multi-actor design with yearly assessments (for a detailed description, see [37]), allowing for a dyadic perspective. Data collection of pairfam started in 2008, and is consistent with the ethical standards for the treatment of human subjects (German Research Foundation, Register Number NE633/10–3). Informed consent was obtained verbally from all participants included in the study. At the time we started our study, data of nine measurement occasions (T0–T8) were available.

### Participants and procedure

*N* = 12,402 anchors (i.e., participants that served as reference persons) from three birth cohorts (1971–1973, 1981–1983 and 1991–1993) participated at T0. Of those, *n* = 6,373 were females, and *n* = 6,027 were males. If anchors in romantic relationships permitted to contact their partners, these were also invited to participate in the study. For the purpose of our gender-controlled dyadic design (i.e., cases were separated by gender), we excluded females and males with same-sex partnerships, and persons who did not reliably indicate their gender. We excluded anchors of the youngest cohort (birth years 1991–1993) because research has shown that adolescent romantic relationships differ qualitatively from romantic relationships later in adulthood [38], so the age groups may not have been readily comparable.

The partners’ self-esteem was only measured from T6 on if the relationship was new, which made the dyadic perspective for continuing relationships impossible after T5. To make our sample more comparable in terms of longitudinal dynamics, we excluded pairs that had been separated at any of the assessment occasions T0–T5. That is, we excluded participants that were single and/or changed their partners within this time span, and couples that were together both at T0 and T5, but indicated a separation phase in between (i.e., at one of the measurement occasions T1–T4). This left a sample with only *continuing couples*. While the exclusion of couples who separated at some time after T0 (*dissolving couples*) was important to appropriately address our research question, it bore the risk of selecting a substantially biased sample. To find out if dissolving and continuing couples differed at T1, we report demographics and descriptive statistics of the study variables for the dissolving couples at T1 in S1 Table.

At T0, anchors’ self-esteem was measured by a personal interview, but since T1, it was assessed via a computer-assisted self-administered interview. As the measurement method had been shown to affect the actual self-esteem measures [6], we decided to exclude T0 from our analysis (compare [9]). We thus conducted all analyses across T1–T5. Little’s MCAR test with all manifest items indicated that data might not be missing at random [χ^2^(17,893) = 21,719.21, *p* < .001]. As the χ^2^-to-*df* ratio of 1.20 revealed, however, this could be due to the large sample size (compare [6]).

At T1, our dataset consisted of *N* = 1,093 continuing female–male couples (i.e., 1,093 females and 1,093 males, respectively) with females reporting a mean age of 32.74 years (*SD* = 5.32, age range: 19–53 years), and males a mean age of 35.57 years (*SD* = 6.03, age range: 21–69 years). For most relationships, anchors and their partners (here, we report the information given by the anchors) indicated to be married/in a civil union (*n* = 796, 72.8%). Mean relationship duration at T1 was 10.65 years (i.e., ten years and eight months, *SD* = 5.69 years, duration range: 1.00–33.58 years). The mean number of children as indicated by the anchors was 1.37, ranging from 0–7 children (*SD* = 1.12). At T1, *n* = 801 (36.6%) of the 2,186 participants had completed the general higher education qualification (“*Allgemeine Hochschulreife*”), qualifying them for studying at university.

### Measures

#### Self-esteem

Self-esteem was assessed via a computer-assisted self-administered interview at all measurement occasions (T1–T5). Three items from the Rosenberg Self-Esteem Scale [39] (“*Sometimes I believe that I’m worthless* (item recoded),” “*I like myself just the way I am*,” and “*All in all*, *I am pleased with myself*”) were answered on a five-point Likert scale ranging from 1 (*not at all*) to 5 (*absolutely*). One item was reversed before the analysis. Coefficient ω and its 95% confidence intervals for females’ self-esteem were .78 [.75, .81], .78 [.75, .81], .78 [0.76, 0.81], .74 [.70, .77], and .79 [.76, .81] at T1–T5, respectively. Coefficient ω estimates for males’ self-esteem were .74 [.71, .77], .74 [.71, .77], .77 [.74, .79], .71 [.66, .75], and .80 [.77, .82] at T1–T5, respectively.

#### Perceived conflict frequency in romantic relationships

Relationship conflict was measured with multiple indicators. First, we used two items of an adapted Conflict Scale from the Network of Relationships Inventory [40] to measure perceived *conflict frequency* within the romantic relationship from both partners’ perspectives from 1 (*never*) to 5 (*always*) at all occasions. The English item translations would be, “*How often do you and [name of current partner] disagree and quarrel*?” and “*How often are you and [name of current partner] annoyed or angry with each other*?” Coefficient ω and its 95% confidence intervals for females’ perceived conflict frequency were .78 [.74, .81], .81 [.78, .83], .79 [.76, .81], .82 [.78, .85], and .81 [.78, .84] at T1–T5, respectively. Coefficient ω estimates of males’ perceived conflict frequency were .76 [.72, .79], .79 [.75, .82], .78 [.75, .81], .80 [.76, .83], and .79 [.76, .81] at T1–T5, respectively.

#### Perceived conflict styles in romantic relationships

In contrast to perceived conflict frequency, we assessed perceived conflict styles of the romantic partner within conflict situations that we hypothesized to be associated to rejection perception. More specifically, we assessed individuals’ perceptions of *their partner’s* behavior within conflict situations via unconstructive and withdrawal behavior. The measures were introduced by the following sentences as documented in the Pairfam Scales Manual (the latest version can be retrieved from **https://www.pairfam.de/dokumentation/scales-manual/**), “*What happens when you have a disagreement with [name of partner]*? *Please indicate how often each of you engaged in the following behaviors*. *When answering*, *please refer to the past six months*,” followed by the introductory question to individuals’ perceptions of their partner’s unconstructive and withdrawal conflict styles, which was, “*How often did your partner engage in any of these behaviors*?”.

We recoded perceived constructive behavior from an adapted Constructive Behavior Scale [41] into *perceived unconstructive behavior* to address the negativity and unwillingness to compromise in relationship conflicts. The English item translations of perceived constructive (unconstructive) behavior in one’s partner as documented in the Scales Manual would be, “*Listen to and ask questions of you in order to understand better* (item recoded)” and “*Endeavor to clarify his or her own position to you* (item recoded).” *Perceived withdrawal behavior* of the partner was assessed via the Withdrawal Scale of the Conflict Resolution Inventory [42]. The English item translations of perceived withdrawal in one’s partner as documented in the Scales Manual would be, “*Remain silent*” and “*Refuse to talk about the subject*.” Both scales were assessed on 5-point scales from 1 (*almost never or never*) to 5 (*very frequently*).

Coefficient ω estimates of females’ perceptions of their male partners’ unconstructive behavior were .72 [.67, .75], .68 [.63, .71], .76 [.72, .79], .72 [.69, .76], and .75 [.71, .78] at T1–T5, respectively. Coefficient ω and its 95% confidence intervals of males’ perceptions of their female partners’ unconstructive behavior were .66 [.61, .70] at T1, .68 [.63, .72] at T2, T3, and T4, and .70 [.65, .74] at T5. Coefficient ω estimates of females’ perceptions of their male partners’ withdrawal were .56 [.51, .61], .61 [.56, .66], .63 [.58, .67], .68 [.63, .72], and .66 [.62, .70]. Coefficient ω and its 95% confidence intervals of males’ perceptions of their female partners’ withdrawal were .61 [.56, .65], .63 [.58, .68], .64 [.59, .68], .68 [.63, .72], and .65 [.59, .69] at T1–T5, respectively.

### Analytical strategy

**Fig 1:**
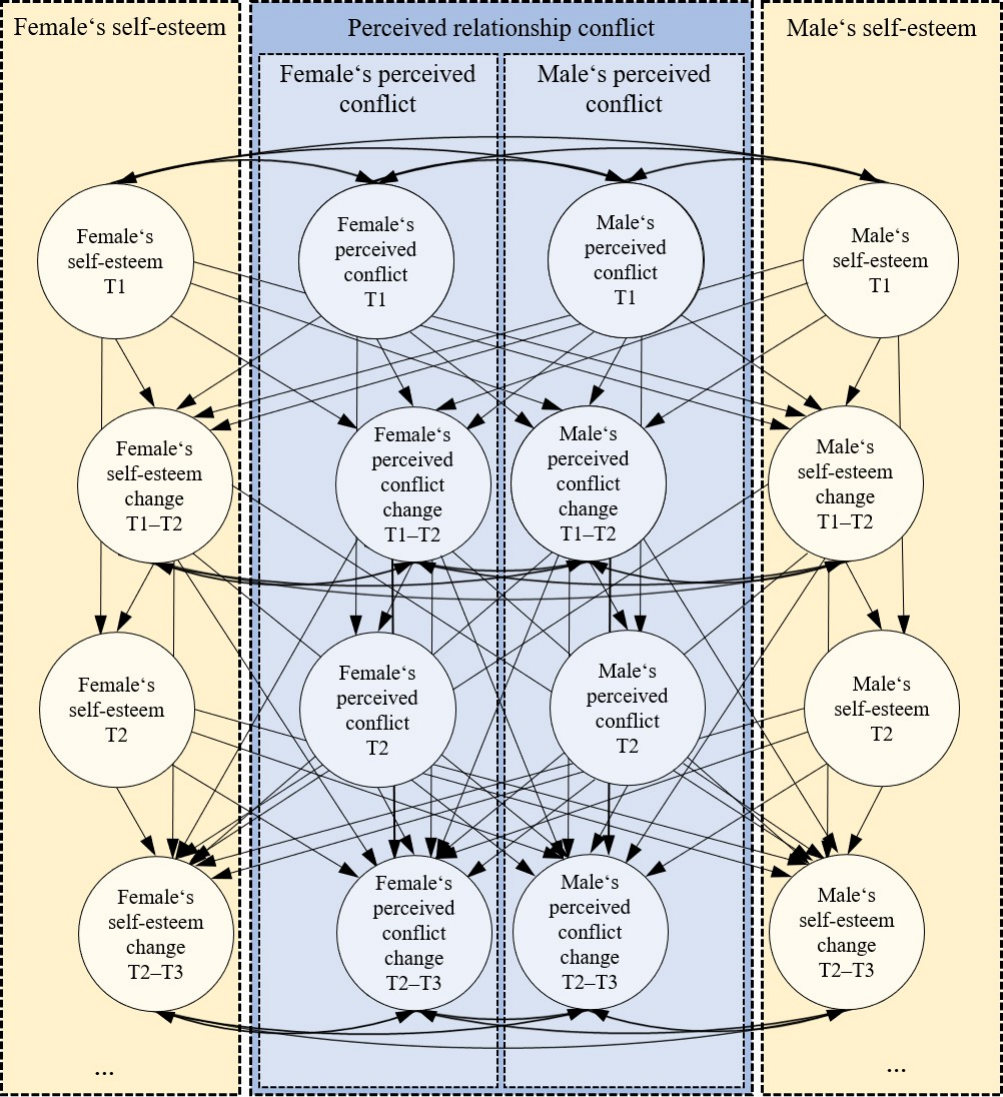
Dyadic extended bivariate latent change model of self-esteem and perceived relationship conflict. For parsimony, we only printed exemplary time points T*n* instead of the five measurement occasions of our model. Measurement models and paths were equated across genders and time periods. Measurement models are omitted for parsimony. Adapted from [6].

We examined transactions between both partners’ self-esteem and aspects of perceived conflict in three separate adapted longitudinal actor–partner (i.e., dyadic) interdependence models [6, 32] (i.e., one for perceived conflict frequency, perceived partner unconstructive behavior, and perceived partner withdrawal, respectively). The use of dyadic bivariate latent change models allowed us to test all of our hypotheses per conflict variable in one model. That is, we were able to assess transactions between self-esteem and aspects of perceived relationship conflict across time and between partners in terms of initial correlations, correlated changes, level-change effects and change-change effects, above and beyond the control for measurement errors, interrelations, and prior variable levels (see Fig 1). Please note that the transactions refer to the factor residuals after controlling for covariates. In the following, we will describe the procedure in more detail.

#### Latent change modeling

To get the most reliable measures, we modeled each anchor’s and partner’s self-esteem and perceived relationship conflict as latent variables, that is, controlling for measurement error [43]. Prior to all analyses, we standardized each variable within gender (except for relationship duration, which we standardized across the total sample). We first established univariate models to individually capture self-esteem and perceived conflict change patterns of females and males in continuing relationships. To assess latent changes, variable levels were decomposed into the initial variable level (i.e., level at T1) and the latent variable changes (i.e., the differences between latent trait levels at two neighboring measurement occasions) [44, 45].

We established strong measurement invariance by equating the latent variable structure, factor loadings, and intercepts for each construct across time in order to reasonably interpret the meaning of their mean-level changes (see S2 Table for descriptive model fits regarding metric and strong measurement invariance) [46]. Moreover, we equated change parameters across time to get the most parsimonious model. Residual variances of each item within a construct were allowed to correlate across time points to account for shared variance that was not accounted for by the latent variables [47].

#### Dyadic bivariate transactions

In a second step, we integrated the univariate models into the dyadic bivariate design. That is, we combined the latent change models for females’ and males’ self-esteem with the respective latent change models for aspects of females’ and males’ perceived conflict. These models allowed us to observe several associations between the constructs under investigation, including initial correlations at T1 within individuals and across partners. Correlated changes, that is, correlations between residuals of change factors within concurrent time intervals, denote the degree to which constructs develop into similar or divergent directions, again both within and across partners.

Level-change effects between two constructs denote how levels in one construct at T1 predict subsequent changes in another construct beyond initial levels. More specifically, while classical crossed-lagged models examine pre-post associations, our approach enabled us to examine the effect of a certain characteristic level on another characteristic’s residual change after ruling out the second characteristic’s initial level. Actor and partner level-change effects from perceived relationship conflict on self-esteem made it possible to test the sociometer hypothesis, while effects of self-esteem on perceived relationship conflict pertain to the self-broadcasting perspective. Last, change-change effects denote how prior changes in one construct predict subsequent changes in the other [34]. We included age and relationship duration as covariates of all effects in our analyses. To yield the most parsimonious model, we equated paths for women and men [6, 48].

Data preparation was done using R version 3.4.4 [49]. The dyadic bivariate latent change models were implemented and run in Mplus version 8 [50], where missing data was handled using FIML [51]. All syntax files can be found at the open science framework (**https://osf.io/9b7mr/**). Combining CFI, RMSEA, and SRMR leads to a good overall model fit criterion for large sample sizes. As a rule of thumb, CFI (comparative fit index) should be > .93, and both RMSEA (root mean square error of approximation) and SRMR (standardized root mean square residual) < .07, respectively [52]. As our model was very complex and we wanted to ensure appropriate per-parameter power, we considered effects with a *p* level of < .05 as statistically significant.

## Results

### Descriptive and univariate change statistics

We did not find any substantial mean-level changes in self-esteem, perceived conflict frequency, and perceived conflict styles (see Table 1). Exceptions were males’ perceptions of their female partners’ unconstructive behavior, increasing across T1–T2 (*d* = .11, 95% CI [.03, .19]) and T3–T4 (*d* = .13, 95% CI [.04, .21]), as well as males’ perceptions of their female partners’ withdrawal, increasing across T3–T4 (*d* = .11, 95% CI [.03, .19]) and decreasing across T4–T5 (*d* = –.10, 95% CI [–.18,–.01]). All variables showed moderate rank-order consistencies for both genders (see Table 1), which indicated individual variability in trait change trajectories. Initial within-construct correlations and correlated changes between partners can be found in S3 Table, within-construct partner level-change and change-change effects in S4 Table.

**Table 1:** Descriptive statistics of the study variables in continuing couples.

	*M* (*SD*)					Cohen’s *d* ^a^ [95% CI]				Stability ^b^ [95% CI]			
Variable	T1	T2	T3	T4	T5	T1→T2	T2→T3	T3→T4	T4→T5	*r*_12_	*r*_23_	*r*_34_	*r*_45_
	*Reported by females*												
Self-esteem	3.81	3.82	3.85	3.85	3.85	.01	.04	.00	.00	.56	.59	.61	.64
	(0.86)	(0.84)	(0.82)	(0.81)	(0.80)	[–.07, .10]	[–.04, .12]	[–.08, .08]	[–.08, .08]	[.52, .60]	[.55, .63]	[.57, .65]	[.60, .67]
Conflict frequency	2.56	2.58	2.59	2.57	2.58	.04	.02	–.04	.02	.66	.64	.68	.70
	(0.61)	(0.64)	(0.62)	(0.63)	(0.63)	[–.04, .12]	[–.07, .10]	[–.12, .04]	[–.06, .10]	[.62, .69]	[.61, .68]	[.64, .71]	[.67, .73]
Uncon-structive behavior	2.49	2.56	2.53	2.59	2.64	.07	–.03	.07	.06	.48	.52	.55	.55
	(0.97)	(0.91)	(0.96)	(0.92)	(0.91)	[–.01, .16]	[–.12, .05]	[–.02, .15]	[–.03, .14]	[.43, .53]	[.47, .56]	[.50, .59]	[.51, .59]
Withdrawal	2.33	2.35	2.29	2.36	2.33	.02	–.06	.08	–.03	.62	.58	.61	.61
	(1.02)	(1.04)	(1.02)	(1.03)	(1.01)	[–.06, .11]	[–.15, .02]	[–.01, .16]	[–.12, .05]	[.58, .65]	[.53, .62]	[.57, .65]	[.57, .65]
*Reported by males*													
Self-esteem	4.03	4.05	4.05	4.03	4.06	.03	.00	–.03	.05	.50	.51	.51	.57
	(0.75)	(0.73)	(0.75)	(0.72)	(0.74)	[–.06, .11]	[–.08, .08]	[–.11, .06]	[–.04, .13]	[.45, .54]	[.46, .56]	[.47, .56]	[.53, .61]
Conflict frequency	2.50	2.47	2.45	2.48	2.44	–.06	–.04	.06	–.08	.62	.63	.63	.63
	(0.62)	(0.63)	(0.59)	(0.62)	(0.61)	[–.14, .03]	[–.12, .05]	[–.03, .14]	[–.16, .01]	[.58, .66]	[.59, .67]	[.59, .67]	[.59, .66]
Uncon-structive behavior	2.35	2.45	2.41	2.52	2.50	.11	–.04	.13	–.02	.42	.41	.46	.48
	(0.84)	(0.86)	(0.84)	(0.84)	(0.81)	[.03, .19]	[–.13, .04]	[.04, .21]	[–.11, .06]	[.36, .47]	[.35, .46]	[.41, .51]	[.43, .53]
Withdrawal	2.08	2.04	2.02	2.11	2.03	–.05	–.02	.11	–.10	.56	.56	.57	.60
	(0.91)	(0.91)	(0.88)	(0.92)	(0.88)	[–.13, .04]	[–.11, .06]	[.03, .19]	[–.18,–.01]	[.52, .60]	[.51, .60]	[.52, .61]	[.55, .64]

*Note*. Conflict frequency = perceived conflict frequency; unconstructive behavior = perceived unconstructive behavior tendencies in partner; withdrawal = perceived withdrawal tendencies in partner. Adapted from [6].

^a^ Within-group repeated-measures raw-score metric, corrected for retest stability. More specifically, we calculated *d* as the difference between the mean levels of two time points, divided by the standard deviation of the respective first time point [53], and corrected for retest stability [54].

^b^ Subscripts denote measurement occasions.

### Transactions between self-esteem and relationship conflict

All models indicated a good fit to the data with RMSEA = .026 [90% CI: .025, .028], CFI = .965, SRMR = .057 for conflict frequency; RMSEA = .026 [90% CI: .024, .028], CFI = .958, SRMR = .053 for partner unconstructive behavior; and RMSEA = .026 [90% CI: .024, .028], CFI = .960, SRMR = .057 for partner withdrawal. Initial correlations between self-esteem and aspects of perceived relationship conflict were negative within individuals (*r* = –.26 to–.12, *p* < .001) and between partners (*r* = –.19 to–.13, *p* < .001, see Table 2) in all cases. Self-esteem changes were also negatively correlated with all aspects of perceived conflict changes within the same time interval, and this also both within individuals (*r* = –.27 to–.14, *p* < .001) and between partners (*r* = –.12, *p* < .001, to–.07, *p* = .001). In our example, decreases in Jenny’s self-esteem would be associated with concurrent increases in James’ perceived conflict frequency. We have to stress, however, that all correlations were in a low range.

**Table 2:** Initial correlations and correlated changes between self-esteem and aspects of perceived relationship conflict.

	Self-esteem ^a^									
	T1		T1→T2		T2→T3		T3→T4		T4→T5	
Conflict variable	*r*	95% CI	*r*	95% CI	*r*	95% CI	*r*	95% CI	*r*	95% CI
	Within individuals (*actor effects*)									
Frequency	–.26	–.31,–.21	–.21	–.25,–.17	–.23	–.28,–.18	–.27	–.32,–.21	–.22	–.26,–.18
Uncon-structive behavior	–.12	–.18,–.07	–.14	–.18,–.10	–.15	–.19,–.11	–.18	–.23,–.13	–.15	–.20,–.11
Withdrawal	–.21	–.28,–.16	–.22	–.27,–.17	–.23	–.28,–.18	–.26	–.32,–.20	–.23	–.28,–.18
	Between partners (*partner effects*)									
Frequency	–.17	–.22,–.12	–.10	–.14,–.05	–.10	–.15,–.06	–.12	–.17,–.07	–.10	–.14,–.06
Uncon-structive behavior	–.13	–.18,–.07	–.07 ^b^	–.11,–.03	–.07 ^b^	–.11,–.03	–.09 ^b^	–.14,–.03	–.07 ^b^	–.12,–.03
Withdrawal	–.19	–.25,–.13	–.10	–.15,–.05	–.10	–.15,–.05	–.11	–.17,–.06	–.10	–.15,–.05

*Note*. Frequency = perceived conflict frequency; unconstructive behavior = perceived unconstructive behavior tendencies in partner; withdrawal = perceived withdrawal tendencies in partner. Coefficients were averaged across sexes due to slight differences in sex-specific variances. Adapted from [6].

^a^ Most correlations were significant at *p* < .001, except for ^b^.

^b^ Correlation was significant at *p* = .001.

#### Level-change effects supporting sociometer perspectives.

Throughout the next paragraphs, we will present level-change effects with regard to sociometer perspectives. We will first describe effects of perceived conflict frequency, before we depict level-change effects of perceived partner conflict styles. We found a small within-person (actor) effect of perceived conflict frequency on subsequent self-esteem changes (β = –.09 [95% CI:–.13,–.05], *p* < .001) above and beyond the initial self-esteem level. In contrast, we did not find a between-person (partner) effect of perceived conflict frequency on changes in self-esteem. That is, while Jenny’s self-esteem would be unaffected by the conflict frequency reported by James, his perception of a high conflict frequency predicted a decrease in his self-esteem.

Significant actor effects of perceived partner unconstructive behavior (β = –.05 [95% CI:–.08;–.02], *p* = .001) and withdrawal (β = –.05 [95% CI:–.08; .02], *p* = .001) on subsequent self-esteem changes supported sociometer perspectives for conflict styles. That is, the more James perceived unconstructive behavior or withdrawal in Jenny, the steeper his self-esteem would decrease in comparison to the average person in our sample. Moreover, we found significant partner effects of perceived partner unconstructive behavior (β = –.04 [95% CI:–.06,–.01], *p* = .017) and perceived partner withdrawal (β = –.07 [95% CI:–.11,–.04], *p* < .001) on subsequent changes in the partner’s self-esteem controlled for their initial self-esteem level. These effects indicated the following: The more James perceived unconstructive or withdrawal tendencies in Jenny, the steeper Jenny’s self-esteem would consequently decrease. All effects were small, explaining only a little proportion of the overall variance in self-esteem changes.

#### Level-change effects supporting self-broadcasting perspectives

Throughout this section, we will first present effects that support self-broadcasting perspectives, before summarizing non-significant effects. We found one small actor effect of self-esteem on subsequent changes in perceived partner unconstructive behavior (β = –.04 [95% CI:–.07;–.01], *p* = .009) after controlling for the initial level of perceived partner unconstructive behavior. That is, the lower James’ initial self-esteem was in comparison to the average person in our sample, the more his perception of Jenny’s unconstructive behavior subsequently increased (or, the less it decreased), above and beyond the control of her prior unconstructive behavior level. The other way round, the higher James’ initial self-esteem was, the more his perception of Jenny’s unconstructive behavior subsequently decreased. Please note that we found no partner effect of self-esteem on subsequent change in perceived partner unconstructive behavior. Put differently, while James’ self-esteem predicted changes in his perception of Jenny’s unconstructive behavior, Jenny’s self-esteem did not predict changes in James’ perception of her unconstructive behavior. Regarding conflict frequency and perceived partner withdrawal, we did not find any support for self-broadcasting perspectives in terms of level-change effects (see Table 3).

**Table 3:** Dyadic latent bivariate level-change and change-change effects.

	Effects of perceived relationship conflict on self-esteem											
	Conflict level on self-esteem change ^a^						Conflict change on self-esteem change ^b^					
Conflict variable	Within individuals (*actor effects*)			Between individuals (*partner effects*)			Within individuals (*actor effects*)			Between individuals (*partner effects*)		
	β	*p*	95% CI	β	*p*	95% CI	β	*p*	95% CI	β	*p*	95% CI
Frequency	–.09	< .001	–.13,–.05	.02	.307	–.02, .06	.05	.022	.01, .10	–.04	.086	–.09, .01
Uncon-structive behavior	–.05	.001	–.08,–.02	–.04	.017	–.06,–.01	–.04	.058	–.08, .01	.02	.296	–.02, .07
Withdrawal	–.05	.001	–.08,–.02	–.07	< .001	–.11,–.04	–.05	.053	–.10, .00	.03	.297	–.02, .08
**Effects of self-esteem on perceived relationship conflict**												
	Self-esteem level on conflict change ^a^						Self-esteem change on conflict change ^b^					
	Within individuals (*actor effects*)			Between individuals (*partner effects*)			Within individuals (*actor effects*)			Between individuals (*partner effects*)		
	β	*p*	95% CI	β	*p*	95% CI	β	*p*	95% CI	β	*p*	95% CI
Frequency	–.02	.300	–.05, .02	.01	.604	–.02, .04	–.06	.009	–.10,–.01	.01	.659	–.03, .05
Uncon-structive behavior	–.04	.009	–.07,–.01	–.01	.352	–.05, .02	.01	.559	–.03, .05	–.03	.205	–.07, .01
Withdrawal	–.02	.375	–.06, .02	–.02	.354	–.06, .02	–.01	.851	–.06, .05	.02	.378	–.03, .08

*Note*. Frequency = perceived conflict frequency; unconstructive behavior = perceived unconstructive behavior tendencies in partner; withdrawal = perceived withdrawal tendencies in partner. For parsimony, standardized coefficients were equated across sexes and time intervals. Adapted from [6].

^a^ T1 on T1 → T2, T2 on T2 → T3, etc.

^b^ T1 → T2 on T2 → T3, T2 → T3 on T3 → T4, etc.

#### Change-change effectss

After having presented level-change effects with regard to sociometer and self-broadcasting perspectives, we will now present significant change-change effects. Please note that we did not subsume change-change effects under the respective perspectives. The reason is that in our opinion, bidirectional longitudinal transactions are best interpreted together. On the actor level, there were two significant change-change effects, both regarding conflict frequency: Increases in self-esteem predicted decreases in perceived conflict frequency (β = –.06 [95% CI:–.10,–.01], *p* = .009). That is, increases in Jenny’s self-esteem during T1–T2 predicted decreases in her reported conflict frequency during T2–T3. Moreover, increases in perceived conflict frequency predicted subsequent increases in self-esteem (β = .05 [95% CI: .01, .10], *p* = .022). That is, Jenny’s notion of an increasing conflict frequency during T1–T2 also predicted increases in her self-esteem during T2–T3. While the effects were very small, this pattern indicates longitudinal transactions between self-esteem and conflict frequency. Apart from that, we found no other change-change effects. That is, there were no hints of bidirectional longitudinal transactions between self-esteem and perceived unconstructive behavior or withdrawal, neither within individuals nor between partners.

## Discussion

The aim of this study was to deepen the knowledge on self-esteem–conflict dynamics in continuing romantic relationships. To that end, we analyzed longitudinal transactions between self-esteem and perceived conflict from both partners’ perspectives, covering a time span of five years. In general, findings indicate that self-esteem and aspects of perceived conflict affected each other within and across time. This was not only true within individuals, but also between partners, indicating a shared negative dynamic within romantic relationships. More specifically, our findings mostly supported the sociometer perspective, with limited support for self-broadcasting perspectives. This conclusion is based on effects of perceived conflict on self-esteem change within and between partners, with only one within-person effect of self-esteem on changes in perceived partner unconstructive behavior.

### Transactions between self-esteem and perceived conflict in continuing couples

With regard to concurrent associations, self-esteem and perceived relationship conflict (i.e., conflict frequency, unconstructive behavior, and withdrawal) were negatively related both within individuals and between partners. Although these results cannot be interpreted in terms of causality, they show a mutual negative dynamic between self-esteem and both conflict frequency and conflict styles, respectively. This is an important finding as this pattern did not only occur within one person, possibly being caused by rater biases, but between partners, too, pointing towards spill-over effects in continuing couples.

#### Support for sociometer perspectives

Investigating level-change effects allowed us to elaborate on the directions of influence. In general, both perceived conflict frequency and conflict styles showed small but significant influences on self-esteem changes, supporting the sociometer perspective. Our findings complement previous results indicating small positive effects of relationship satisfaction aspects on both partners’ self-esteem changes [6]. In sum, our findings suggest that sociometer perspectives might be valid for both perceived conflict frequency and conflict styles.

While conflict frequency had a within-person effect on self-esteem, conflict styles showed both within-person and between-person effects. Turning to the within-person findings, we found negative actor effects of perceived conflict frequency and conflict styles on subsequent self-esteem changes. That is, the more conflict individuals perceived in their relationship and the less functional their perceived partner behavior was during these conflicts, the steeper was their decrease in self-esteem thereafter, after controlling for previous self-esteem levels. Although these effects were small, they might indicate that individuals experience both conflict frequency in romantic relationships and dysfunctional partner behavior during conflicts as indicators of social rejection [19, 20, 22].

Moreover, we observed partner effects of perceived conflict styles on changes in self-esteem. Referring to our example of Jenny and James, James’ perception of Jenny showing high levels of unconstructive behavior and withdrawal during conflict situations affected stronger decreases in Jenny’s self-esteem. These findings are in line with Srivastava and Beer [23], who reported that insecure attachment behavior predicted lower self-evaluations in the same individuals. They also conceptually converge with previous findings on negative effects of perceived or feared disapproval and rejection on the level of self-esteem [21], although no spill-over effects between partners were tested in the previous study. One explanation of these findings might look like this: James perceives and disapproves of Jenny’s dysfunctional behavior during conflicts. Subsequently, he becomes less satisfied with their relationship [20]. Jenny, in turn, interprets James’ dissatisfaction as rejection, leading her self-esteem to decrease [21]. In general, it might be true that the perception of social rejection affects both persons involved, that is, the person showing rejection behavior and the person perceiving it. As our study design does not allow to uncover underlying mechanisms, this possibility should be addressed in future studies.

Originally, the sociometer perspective was developed for state self-esteem [22]. That is, self-esteem is expected to fluctuate in close timely proximity after social rejection situations. While a number of studies supported this notion, many of them based their conception of self-esteem on the Rosenberg Self-Esteem Scale [19, 20, 25], which resembles a *trait* approach to self-esteem [55]. As our study investigated effects of perceived conflict on self-esteem across one-year time intervals, it seems warranted to infer theoretical and methodological implications for a trait perspective on the sociometer concept. In fact, Leary et al. [22] acknowledge that experiencing real or imagined rejection over a long period of time may lower trait self-esteem, that is, the “average resting position of the ‘indicator needle’ on the person’s sociometer” [22, p. 527]. Our findings imply the following: Social influences in terms of conflict on trait self-esteem seem to represent more than only short-term phenomena. Rather, they can impact the fairly stable trait self-esteem over the course of several months. What remains unexplained are the underlying processes.

Whole trait theory [56] was originally developed for the Big Five traits, but might prove useful in this regard. This theory posits that social-cognitive mechanisms can explain differential behavior in situations (i.e., distributions in states). In short, despite situational deviations, changes in general social cognitions and thus changes in average general behavioral tendencies (i.e., state aggregates) can alter the average trait level [56]. In our example, James’ state self-esteem might decrease after he feels rejected by Jenny. Manifesting a general social cognition of Jenny as rejecting may increase the frequency of these state reactions (i.e., decreased state self-esteem) in multiple situations, eventually lowering James’ trait self-esteem.

Besides, our results indicate that negative conflict behavior was not a within-person experience only, but seemed to have consequences for both partners. Couple consultants, for example, should bear in mind that one partner’s communication and interaction behavior possibly affect both individuals’ self-esteem, which in turn may impact different areas of their lives–for example, their mental and physical health [57, 58]. Our study emphasizes the importance of assessing both individuals’ perceptions in dyadic interactions.

#### Support for self-broadcasting perspectives

While there were no actor and partner level-change effects regarding conflict frequency and withdrawal, we found one small actor effect that supports self-broadcasting perspectives regarding unconstructive behavior. More specifically, a person’s self-esteem predicted changes in their perception of their partner’s unconstructive behavior, indicating that lower levels of self-esteem affected an increase in perceived unconstructive behavior. Murray and colleagues [21] had found the same effect on conflict–negativity and combined destructive conflict styles. So, while our study in part supports the authors’ [21] findings, our data also highlight the idea that self-esteem only affects specific dysfunctional conflict styles.

Interestingly, Murray and colleagues [20] found a spill-over effect of self-esteem related sensitivity to rejection on the partner’s relationship satisfaction, pointing towards between-person dynamics for this construct. While we did not find between-person effects supporting self-broadcasting perspectives, the within-person effect of low self-esteem on increases of perceived unconstructive partner behavior in our sample might indicate a similar process. More specifically, low self-esteem individuals might show behavior that their partners do not approve of, making them less satisfied over time [20]. Possibly triggered by this dissatisfaction, their partners behave less constructive during conflicts [compare 21]. This change in behavior would then be noticed by the low self-esteem individuals, manifesting in their increased perceived partner unconstructive behavior.

More generally, the pattern of results with regard to self-broadcasting perspectives was inconsistent. This tends to be in line with recent studies [6, 31, 48] reporting only small self-broadcasting effects for relationship satisfaction [59] and diverging effects for other indicators of relationship quality. Possibly, self-broadcasting effects might only hold in terms of certain relationship aspects, but not in terms of others. If this speculation holds true, it remains yet to be explained, however, which processes enforce these differences. To clear up this uncertainty, future research would need to test differences between relationship dynamics systematically with the help of multiple, comparably objective measures.

#### Change-change effects

With regard to change-change effects, we found an interesting pattern for self-esteem and conflict frequency at the actor level. At first glance, the change-change effects occurred in both directions, implying support for sociometer as well as self-broadcasting perspectives. However, the direction of effects was less straightforward. In line with self-broadcasting perspectives, decreases in self-esteem predicted subsequent increases in perceived conflict frequency. In contrast, decreases in perceived conflict frequency were aligned to subsequent decreases in self-esteem (that is, increases in conflict frequency were aligned to subsequent increases in self-esteem). This finding is opposite to the predictions of the sociometer perspective. Possibly, increasing conflict frequency might not only have negative implications for individuals’ self-concepts. Jenny and James might find that they argue more frequently now than one year ago. However, they also observe a constructive culture of debate, making it possible to not only argue over different points of view, but to come up with solutions. Observing this dynamic in turn might let their self-esteem increase as a function of perceived self-efficacy for jointly solving problems. This interpretation is, however, mere speculation and needs to undergo further hypothesis testing.

To explain this finding from a statistical perspective, it might be helpful to bear in mind the other findings. The first information that sticks out were the high initial levels of self-esteem. Moreover, decreases in perceived conflict frequency were accompanied by increases in self-esteem during the same time span (correlated changes). Combining the negatively correlated changes between self-esteem and conflict frequency within the same time interval with high initial self-esteem levels, this might lead to inflated self-esteem measures at T2 for the individuals who reported decreases in conflict frequency across T1–T2. That is, while James’ perceived conflict frequency, for example, decreased, his self-esteem increased across T1–T2 up until the highest possible value. And although it would be plausible to assume that his self-esteem would increase even more across T2–T3, this is not possible. What follows is that his self-esteem can only decrease or not change after T2, leading to decreases in self-esteem between T2–T3 after decreases in conflict frequency between T1–T2. Assuming a highly probable regression to the mean, it is in fact very likely that his self-esteem decreases in this scenario, leading to the confusing pattern from above. In contrast, while James’ self-esteem increases between T1–T2, this perceived conflict frequency decreases during this time span, possibly and in line with the average mean levels in conflict frequency. In this case, it is possible that his perceived conflict frequency decreases further between T2–T3, leading to support of the self-broadcasting perspective. So, while these findings seem to be exciting, they require further investigation to rule out possible statistical artefacts due to ceiling effects of our sample’s high average self-esteem levels. In total, they still confirm the notion that romantic relationships are a meaningful context for self-esteem development, which is sensitive to perceived relationship conflict.

### Limitations and future directions

This study has limitations. First, we only investigated dyads in continuing young adult female–male relationships with an initial mean relationship duration of eleven years who stayed together for at least five more years. As low self-esteem has been found to predict relationship break-up [9], different patterns of dynamic transactions seem plausible in dissolving couples. Although previous work does not suggest that conflict functions as a mediator between self-esteem and break-up [60], it might still be worthwhile to examine self-esteem–conflict transactions in couples destined to break up (compare [61]).

Second, our study design followed one-year time intervals, and did not allow to uncover mechanisms behind negative relationship dynamics in a suitable way. Our findings imply that one-year time intervals seem to be appropriate to capture a trait approach on self-esteem changes. However, we cannot conclude from our findings if and how state and trait self-esteem may interact with each other in building these effects. According to the authors of Whole trait theory [56], several requisites must be met in order for an individual to produce a specific behavior in any given situation. Amongst others, the situation must be interpreted as relevant and favorable, and the individual must be motivated to reach or prevent certain end-states [56]. In the following, we will outline how future studies could be construed to explicitly test these assumptions. For study construction that allows testing Whole trait theory regarding transactions between self-esteem and perceived conflict, we would need a combination of meticulous and frequent short-term assessments during conflict situations and a long-term approach. The TESSERA framework [62] suggests decomposing complex social situations into smaller units. This option can be applied to relationship situations that trigger and follow conflict behavior. Although the framework explicitly includes reactions from others, it is best applied to *one* individual. Integrating this framework into the PERSOC framework [63] might help disentangle the micro-processes underlying the sociometer (and possible self-broadcasting) effects that we found in our study from *both* partners’ perspectives in dyads. To assess self-esteem dynamics during and after conflict situations, it seems especially promising to assess the following parameters besides perceived partner conflict behavior, state self-esteem, and trait self-esteem: interpreting the situation (e.g., as rejecting), and pursuing inclusion [see 56 for more information]. We feel that this is an exciting approach to apply these measures with the goal to better understand long-term self-esteem dynamics. This way, it might be possible to uncover the mechanisms behind conflict situations that lead to self-esteem changes.

Third, this study focused on transactions between self-esteem and negative relationship aspects such as conflict, but did not integrate positive relationship aspects, such as satisfaction. There is a great deal of research showing interrelations between self-esteem and relationship satisfaction [6, 20, 25]. Some studies also suggest associations between conflict and relationship satisfaction [14, 15]. However, there is not much research to date that has investigated the role of self-esteem in this association. Future research might profit from including self-esteem, relationship conflict, and satisfaction in one longitudinal model to systematically disentangle their interrelations. This might especially be interesting as we found strong effects of perceived conflict on self-esteem changes, but less support for the opposite direction.

Relationship satisfaction and self-esteem seem to influence each other more mutually [6]. Some studies even suggest that self-esteem is a better predictor of satisfaction than vice versa [57]. As we found only one self-broadcasting effect on perceived conflict changes, perceived relationship conflict might predict the other constructs, with self-esteem being the mediating variable that, subsequently, explains decreasing relationship satisfaction. This is of course only speculation and should be investigated systematically by future studies. Moreover, it seems likely that relationships high in frequent and dysfunctional conflict are less stable than those low in these measures [16, 17, 64]. In line with this, Finn and colleagues [61] found a higher baseline conflict frequency and a lower baseline satisfaction in couples who later dissolve than in continuing couples. Moreover, the authors have reported a stronger increase in conflict frequency and a stronger decrease in satisfaction in later dissolving couples. While our findings support the notion that dissolving couples show lower initial self-esteem levels and higher conflict levels, conflict and conflict styles alone do not seem to be good predictors of relationship stability [9, 60].

Fourth, perceived conflict frequency was assessed annually using self-reports, while conflict styles were assessed annually with the use of perceived reports about the partner. Although we used partner reports to yield a more objective perspective on the individuals’ actual behavior [65, 66], we cannot know if perceived conflict frequency and conflict styles were objective measures. Nevertheless, we controlled for prior levels of all constructs during investigating their longitudinal associations, which resembles a control of at least some confounding measures.

Further, we only investigated young to midlife adults’ self-esteem–conflict transactions. It might be reasonable that younger or older couples differ regarding their relationship dynamics. Finally, other authors have established a *p* level of < .01 [35, 67, 68] to account for the risk of inflating Type-I errors in multivariate analyses [69, 70]. However, such error controlling procedures might be overly conservative [71], especially in very complex models. We thus considered effects with a *p* level of < .05 as statistically significant. Although some effects were significant at *p* < .01, all effects were very small. Thus, although true transactions between self-esteem and perceived conflict are probable, they might still not be important in everyday life.

## Conclusions

Our study aimed at investigating transactions between self-esteem and perceived conflict in continuing romantic couples from the perspectives of both partners. We found support for sociometer perspectives, although effect sizes were small. As indicated by subsequent self-esteem decreases, perceived conflict frequency and perceived partner conflict styles might function as indicators of social rejection. Importantly, we showed that partner-rated dysfunctional behavior during conflicts subsequently predicted an individual’s self-esteem changes. Put differently, if one partner reports dysfunctional behavior in the other partner during conflicts, the second partner’s self-esteem subsequently decreased. This implies that social situations are important in order to understand the dynamics of self-esteem changes. In contrast to studies focusing on relationship satisfaction, we only found little support for self-broadcasting perspectives. We thus conclude that dynamics between self-esteem and perceived relationship conflict might differ from transactions with relationship satisfaction in a way that their dynamics may not be parallel to each other. Our findings imply that both relationship satisfaction and conflict should be considered in future studies investigating self-esteem-relationship transactions in order to consolidate findings. Moreover, future studies should investigate thoughts and feelings within conflict situations to derive potential mechanisms underlying these effects.

## Supporting information

S1 Table Comparing continuing to dissolving couples at T1. *Note*. Conflict frequency = perceived conflict frequency; unconstructive behavior = perceived unconstructive behavior tendencies in partner; withdrawal = perceived withdrawal tendencies in partner. Adapted from [6]. ^a^ *n* = 1,093. ^b^ *n* = 811. ^c^ Mean-level differences between two independent groups of different sizes. Continuing couples = reference group.
(DOCX)

S2 Table Establishing strong measurement invariance within univariate models. *Note*. ^a^ Due to small variances of the respective second indicators and problems involving the covariance matrix, we set the correlation between the second indicators of the fifth and sixth item to zero. This did not alter the model fit.
(DOCX)

S3 Table Initial correlations and correlated changes between individuals (partner effects) within self-esteem and aspects of relationship conflict. *Note*. Conflict frequency = perceived conflict frequency; unconstructive behavior = perceived unconstructive behavior tendencies in partner; withdrawal = perceived withdrawal tendencies in partner. Coefficients were averaged across sexes due to slight differences in sex-specific variances. Adapted from [6].
(DOCX)

S4 Table Level-change and change-change effects within self-esteem and perceived relationship conflict. *Note*. Frequency = perceived conflict frequency; unconstructive behavior = perceived unconstructive behavior tendencies in partner; withdrawal = perceived withdrawal tendencies in partner. For parsimony, unstandardized coefficients were equated across sexes and across time intervals. Adapted from [6]. ^a^ T1 on T1 → T2, T2 on T2 → T3, etc. ^b^ T1 → T2 on T2 → T3, T2 → T3 on T3 → T4, etc.
(DOCX)
