# Supplementary material for: Transactions between self-esteem and perceived conflict in romantic relationships: A 5-year longitudinal study
Source: PLoS One. 2021 Apr 12;16(4):e0248620. doi: 10.1371/journal.pone.0248620 (PMC8041199; doi:10.1371/journal.pone.0248620)
Supplement: S1 Table — Note. Conflict frequency = perceived conflict frequency; unconstructive behavior = perceived unconstructive behavior tendencies in partner; withdrawal = perceived withdrawal tendencies in partner. Adapted from [6]. a n = 1,093. b n = 811. c Mean-level differences between two independent groups of different sizes. Continuing couples = reference group. (DOCX) [file pone.0248620.s001.docx]

**Comparing continuing to dissolving couples**

Of course, our sample was biased in the way that only continuing couples’ interactions were followed. While the exclusion of couples who separated after T1 (*dissolving couples*) was important to appropriately address our research question, it bore the risk of selecting a substantially biased sample. To find out if couples who separated and continuing couples differed at T1, we report demographics and descriptive statistics of the study variables for the dissolving couples at T1 here, too, and compare them to the continuing couples’ characteristics. As for the continuing couples, we excluded same-sex partnerships, persons who did not reliably indicate their gender, anchors of the youngest cohort (birth years 1991–1993), as well as relationships with a duration of less than 12 months at T1.

For *n* = 811 of the remaining couples, one or both partners indicated that they had separated between at least one of the occasions T1–T2, T2–T3, T3–T4, and T4–T5. Regarding age, this sample did not differ much from the continuing couples. On average, females were 31.98 years (*SD* = 5.71, age range: 18–57 years) and males 34.71 years (*SD* = 5.99, age range: 24–63 years) old at T1. Mean relationship duration of dissolving couples was 9.99 years (i.e., ten years, *SD* = 5.78 years, duration range: 1.00–29.75 years) at T1. Again, they did not systematically differ from the continuing couples. Similar to the sample of continuing couples (72.8%), the majority (*n* = 540, 66.6%) of anchors indicated to be married/in a civil union. The mean number of children was also similar to that of the continuing couples (*M* = 1.29, *SD* = 1.19), also ranging from 0–7. And with 31.9% (*n* = 518 of 1,622 participants), later dissolving couples had completed the general higher education qualification (“*Allgemeine Hochschulreife*”) comparably often as continuing couples (36.6%).

Regarding the study variables, we found an interesting pattern. While self-esteem at T1 did not differ between the samples, conflict frequency of later dissolving couples was higher than conflict frequency of continuing couples (S1 Table). Unconstructive behavior did not significantly differ for these manifest variables, although confidence intervals suggested a trend towards more unconstructive behavior in dissolving couples, especially for females’ unconstructive behavior reported by males. Withdrawal showed the same trend, which was significant for males, but not for females. To conclude, trends implied differences in both self-esteem and relationship characteristics between the samples, although broad confidence intervals made it difficult to detect statistically significant effects [71].

**S1 Table. Comparing continuing to dissolving couples at T1.**

|  | ***M* (*SD*)** | |  |  |
| --- | --- | --- | --- | --- |
| **Couples** | **Continuing** ^a^ | **Dissolving** ^b^ |  | **Cohen’s *d*** ^c^ **[95% CI]** |
|  | *Reported by females* | | | |
| Self-esteem | 3.81 (0.86) | 3.75 (0.94) |  | –.07 [–.16, .02] |
| Conflict frequency | 2.56 (0.61) | 2.67 (0.67) |  | .17 [ .08, .26] |
| Uncon-structive behavior | 2.49 (0.97) | 2.54 (1.01) |  | .05 [–.04, .14] |
| Withdrawal | 2.33 (1.02) | 2.42 (1.11) |  | .09 [–.01, .18] |
|  | *Reported by males* | | | |
| Self-esteem | 4.03 (0.75) | 3.99 (0.81) |  | –.05 [–.14, .04] |
| Conflict frequency | 2.50 (0.62) | 2.58 (0.66) |  | .13 [ .04, .22] |
| Uncon-structive behavior | 2.35 (0.84) | 2.43 (0.90) |  | .09 [ .00, .18] |
| Withdrawal | 2.08 (0.91) | 2.19 (0.97) |  | .12 [ .03, .21] |

*Note.* Conflict frequency = perceived conflict frequency; unconstructive behavior = perceived unconstructive behavior tendencies in partner; withdrawal = perceived withdrawal tendencies in partner. Adapted from [6].

^a^ *n* = 1,093.

^b^ *n* = 811.

^c^ Mean-level differences between two independent groups of different sizes. Continuing couples = reference group.
