# Supplementary material for: Transactions between self-esteem and perceived conflict in romantic relationships: A 5-year longitudinal study
Source: PLoS One. 2021 Apr 12;16(4):e0248620. doi: 10.1371/journal.pone.0248620 (PMC8041199; doi:10.1371/journal.pone.0248620)
Supplement: S2 Table — Note. a Due to small variances of the respective second indicators and problems involving the covariance matrix, we set the correlation between the second indicators of the fifth and sixth item to zero. This did not alter the model fit. (DOCX) [file pone.0248620.s002.docx]

**S2 Table. Establishing strong measurement invariance within univariate models.**

|  | Descriptive model fits | | | | | |  |  | | |  |
| --- | --- | --- | --- | --- | --- | --- | --- | --- | --- | --- | --- |
|  | Metric measurement invariance | | | Strong measurement invariance | | |  | Wald tests of parameter constraints | | | |
| Model | RMSEA [90% CI] | CFI | SRMR | RMSEA [90% CI] | CFI | SRMR |  | Δχ² | *df* | *p* | |
| Self-esteem | .037 [.034, .040] | .954 | .072 | .037 [.034, .039] | .954 | .072 |  | 0.16 | 8 | >.999 | |
| Conflict frequency | .025 [.021, .030] | .988 | .025 | .025 [.020, .029] | .989 | .025 |  | 0.04 | 4 | >.999 | |
| Unconstructive behavior | .030 [.026, .034] | .972 | .034 | .029 [0.25, .034] | .973 | .034 |  | 0.05 | 4 | >.999 | |
| Withdrawal | .024 [.019, .028] | .985 | .031 ^a^ | .023 [.018, .028] | .985 | .031 ^a^ |  | 0.03 | 4 | >.999 | |
| *Note.*  ^a^ Due to small variances of the respective second indicators and problems involving the covariance matrix, we set the correlation between the second indicators of the fifth and sixth item to zero. This did not alter the model fit. | | | | | | | | | | |  |
