# Supplementary material for: Transactions between self-esteem and perceived conflict in romantic relationships: A 5-year longitudinal study
Source: PLoS One. 2021 Apr 12;16(4):e0248620. doi: 10.1371/journal.pone.0248620 (PMC8041199; doi:10.1371/journal.pone.0248620)
Supplement: S3 Table — Note. Conflict frequency = perceived conflict frequency; unconstructive behavior = perceived unconstructive behavior tendencies in partner; withdrawal = perceived withdrawal tendencies in partner. Coefficients were averaged across sexes due to slight differences in sex-specific variances. Adapted from [6]. (DOCX) [file pone.0248620.s003.docx]

**Initial correlations and correlated changes within self-esteem and relationship conflict**

Initial correlations between partners regarding self-esteem were low (*r* = .13, *p* < .001), and so were (marginally) correlated changes (*r* = .04 to .07, *p* ≤ .060; see S3 Table). Correlations of perceived conflict frequency were high at T1 (*r* = .62, *p* < .001), and correlated changes between partners indicated a mutual development in perceived conflict frequency (*r* = .28 to .33, *p* < .001). Correlations of perceived partner unconstructive behavior at T1 (*r* = .22, *p* < .001) and changes in perceived partner unconstructive behavior were small, yet significant (*r* = .09 to .11, *p* ≤ .011), possibly indicating mutual reinforcement regarding changes in (perceptions of) unconstructive behavior tendencies within a relationship. For withdrawal, we found a similar pattern: Despite no initial correlation, significant (yet small) positive correlations between partners’ subsequent changes in their perception of this behavior in their partner (*r* = .12 to .13, *p* = .016) possibly indicated mutual reinforcement of (perceived) withdrawal tendencies. Please note that effects did not substantially differ across time intervals.

**S3 Table. Initial correlations and correlated changes between individuals (partner effects) within self-esteem and aspects of relationship conflict.**

|  | T1 | | | |  | | T1→T2 | | | |  | | T2→T3 | | | |  | T3→T4 | | |  | T4→T5 | | | |
| --- | --- | --- | --- | --- | --- | --- | --- | --- | --- | --- | --- | --- | --- | --- | --- | --- | --- | --- | --- | --- | --- | --- | --- | --- | --- |
| **Vari-able** | *r* | *p* | 95% CI |  | | *r* | | *p* | 95% CI |  | | *r* | | *p* | 95% CI |  | | *r* | *p* | 95% CI |  | *r* | *p* | 95% CI | |
|  | **Within the model of perceived conflict frequency** | | | | | | | | | | | | | | | | | | | | | | | |  |
| Est. | .13 | <.001 | .06; .20 |  | | .04 | | .059 | –.00; .09 |  | | .05 | | .060 | –.00; .10 |  | | .06 | .060 | –.00; .13 |  | .05 | .060 | –.00; .10 | |
| Freq. | .62 | <.001 | .56; .68 |  | | .29 | | <.001 | .21; .37 |  | | .33 | | <.001 | .24; .42 |  | | .33 | <.001 | .24; .42 |  | .28 | <.001 | .21; .36 | |
|  | **Within the model of perceived unconstructive behavior in partner** | | | | | | | | | | | | | | | | | | | | | | | |  |
| Est. | .13 | <.001 | .06; .20 |  | | .05 | | .041 | .00; .09 |  | | .05 | | .041 | .00; .10 |  | | .07 | .041 | .00; .13 |  | .05 | .041 | .00; .10 | |
| Un-const. | .22 | <.001 | .13; .30 |  | | .09 | | .011 | .02; .17 |  | | .10 | | .011 | .02; .17 |  | | .11 | .010 | .03; .19 |  | .10 | .011 | .02; .18 | |
|  | **Within the model of perceived withdrawal in partner** | | | | | | | | | | | | | | | | | | | | | | | |  |
| Est. | .13 | <.001 | .06; .20 |  | | .05 | | .028 | .01; .10 |  | | .06 | | .029 | .01; .10 |  | | .07 | .028 | .01; .14 |  | .06 | .028 | .01; .11 | |
| With. | .06 | .249 | –.04; .16 |  | | .13 | | .016 | .02; .23 |  | | .12 | | .016 | .02; .22 |  | | .12 | .016 | .02; .21 |  | .12 | .016 | .02; .21 | |

*Note.* Est. = self-esteem, freq. = perceived conflict frequency, unconst. = perceived unconstructive behavior tendencies in partner, with. = perceived withdrawal tendencies in partner. Coefficients were averaged across sexes due to slight differences in sex-specific variances. Adapted from [6].
