# Supplementary material for: Transactions between self-esteem and perceived conflict in romantic relationships: A 5-year longitudinal study
Source: PLoS One. 2021 Apr 12;16(4):e0248620. doi: 10.1371/journal.pone.0248620 (PMC8041199; doi:10.1371/journal.pone.0248620)
Supplement: S4 Table — Note. Frequency = perceived conflict frequency; unconstructive behavior = perceived unconstructive behavior tendencies in partner; withdrawal = perceived withdrawal tendencies in partner. For parsimony, unstandardized coefficients were equated across sexes and across time intervals. Adapted from [6]. a T1 on T1 → T2, T2 on T2 → T3, etc. b T1 → T2 on T2 → T3, T2 → T3 on T3 → T4, etc. (DOCX) [file pone.0248620.s004.docx]

**Level-change effects and change-change effects within constructs**

We found small positive effects of an individual’s self-esteem level on their partner’s subsequent self-esteem changes within the model of perceived conflict frequency (β = .03, *p* = .039), but not in the models of perceived partner’s dysfunctional conflict styles (compare S4 Table), pointing to very small shared partner dynamics in self-esteem. Besides, the relatively more conflict one partner perceived at one time point and the more unconstructive behavior they reported, the more the other partner’s perceived conflict frequency increased in the subsequent time interval and the more their partner’s perception of unconstructive behavior increased, respectively (partner effects; besides positive correlated changes, see S3 Table). Put differently, Jenny’s perceived conflict frequency, for example, predicted subsequent changes in James’ perceived conflict frequency (β =.14, *p* < .001). Moreover, the more unconstructive behavior Jenny reported about James at one time point, the more James’ perception of Jenny’s unconstructive behavior increased during the subsequent time interval (β = .06, *p* = .001). Please note that changes in the partners’ perceived unconstructive behavior during the same time interval were also correlated with each other (yet very small, see S3 Table). Interestingly, we did not find any partner level-change effects regarding withdrawal. However, S3 Table indicates that withdrawal was mutually reinforced between partners – however, more within the same time interval, and not so much across time points. No longitudinal change-change effects were found between partners. Please note that we do not interpret actor effects within constructs.

**S4 Table. Level-change and change-change effects within self-esteem and perceived relationship conflict.**

|  | Effects within self-esteem | | | | | | | | | | | | | | |  |
| --- | --- | --- | --- | --- | --- | --- | --- | --- | --- | --- | --- | --- | --- | --- | --- | --- |
|  | Self-esteem level on self-esteem change ^a^ | | | | | | |  | Self-esteem change on self-esteem change ^b^ | | | | | | | |
| Model of | Within individuals  (*actor effects*) | | |  | Between individuals  (*partner effects*) | | |  | Within individuals  (*actor effects*) | | |  | Between individuals  (*partner effects*) | | | |
|  | β | *p* | 95% CI |  | β | *p* | 95% CI |  | β | *p* | 95% CI |  | β | *p* | 95% CI | |
| Frequency | –.37 | <.001 | –.40, –.34 |  | .03 | .039 | .00, .06 |  | –.25 | <.001 | –.29, –.22 |  | –.01 | .438 | –.05, .02 | |
| Uncon-structive behavior | –.37 | <.001 | –.40, –.34 |  | .03 | .054 | –00, .06 |  | –.27 | <.001 | –.30, –.23 |  | –.01 | .610 | –.04, .02 | |
| Withdrawal | –.37 | <.001 | –.40, –.33 |  | .02 | .143 | –.01, .06 |  | –.27 | <.001 | –.31, –.23 |  | –.01 | .603 | –.04, .03 | |
|  | **Effects within perceived relationship conflict** | | | | | | | | | | | | | | |  |
|  | Conflict level on conflict change ^a^ | | | | | | |  | Conflict change on conflict change ^b^ | | | | | | | |
|  | Within individuals  (*actor effects*) | | |  | Between individuals  (*partner effects*) | | |  | Within individuals  (*actor effects*) | | |  | Between individuals  (*partner effects*) | | | |
|  | β | *p* | 95% CI |  | β | *p* | 95% CI |  | β | *p* | 95% CI |  | β | *p* | 95% CI | |
| Frequency | –.27 | <.001 | –.32, –.22 |  | .14 | <.001 | .10, .19 |  | –.35 | <.001 | –.41, –.29 |  | –.01 | .605 | –.07, .04 | |
| Uncon-structive behavior | –.27 | <.001 | –.31, –.23 |  | .06 | .001 | .02, .10 |  | –.37 | <.001 | –.43, –.31 |  | –.02 | .512 | –.06, .03 | |
| Withdrawal | –.18 | <.001 | –.23, –.13 |  | .04 | .087 | –.01, .08 |  | –.31 | <.001 | –.39, –.23 |  | .05 | .187 | –.02, .11 | |

*Note.* Frequency = perceived conflict frequency; unconstructive behavior = perceived unconstructive behavior tendencies in partner; withdrawal = perceived withdrawal tendencies in partner. For parsimony, standardized coefficients were equated across sexes and time intervals. Adapted from [6].

^a^ T1 on T1 → T2, T2 on T2 → T3, etc.

^b^ T1 → T2 on T2 → T3, T2 → T3 on T3 → T4, etc.
